# Editorialets

**Published:** 1910-06

**Authors:** 


### Editorialets.

---

MARRIED at San Marcos, Texas, May 25 (ult.), Dr. J. M. Hons to Mrs. Willie Swann Foster, both of San Marcos.

---

THE *Oklahoma Medical News Journal*, of Oklahoma City, published a special headache number for May. Articles are furnished by the following: Dr. S. Grover Burnett, Neurologist, of Kansas City, Mo.; Dr. W. T. Salmon, Ophthalmologist, of Oklahoma City, Okla.; Dr. Geo. A. Still, of the original Still School (Osteopathic), of Kirkville, Mo.; and Dr. Alva A. Gregory, head of the Palmer-

Gregory Chiropractic College of Oklahoma City, Okla. This is an unusual and interesting issue and will be sent to any address upon receipt of 25 cents.

---

THE MAY *American Journal of Clinical Medicine,* Chicago, was "The New Graduates' Number," filled with matter of especial interest to them. The June number will be a tuberculosis edition. Sample copies on request. They are whizzers.

---

THE STATE BOARD OF MEDICAL EXAMINERS will meet in the hall of the House of Representatives in Austin, June 28 (inst.). Applicants for examination should file application with the secretary not later than June 15. Dr. J. D. Osborn, of Cleburne, is President, and Dr. M. E. Daniel, of Honey Grove, Texas, is Secreretary.

---

FOR SALE.—Interest in a new, thoroughly equipped hospital that cost $115,000 that is running on a paying basis, can be purchased by the right kind of a man. In a city of 50,000 population in one of the South Atlantic States. Very fine opportunity for man of ability with some means. For further information apply to R., care of this journal.

---

DEAR DR. DANIEL: A line or two from a very busy man who sometimes is remiss in correspondence always carries a warm spot in his heart for old Texas friends, especially for the "Red Back." I should miss it, for it comes with the spicy aroma of F. E. D. always welcome and refreshing.

San Francisco, Cal., May 7, 1910. A. F. SAMPSON, M. D.

---

DEAR DR. DANIEL: I am enclosing my check for renewal. Do not allow my subscription to expire, for I glory in the "Red Back." I glory in your pluck, and especially in your endeavors to prevent the medical profession becoming abject slaves of the "Chicago Gang," who are trying by despotic measures to bring the masses of the profession into abject servitude.

Respectfully yours,

DR. W. CHAS. COVEY,

Hudsonville, Ottawa county, Michigan.

THE AMERICAN SOCIETY OF TROPICAL MEDICINE will hold its seventh annual meeting in St. Louis, at St. Louis University Medical College, June 11, inst. Gorgas is President.

The Council recommends the election of the following officers:

President—William Sidney Thayer, Baltimore.

Vice-Presidents—Rudolph Matas, New Orleans; Judson Daland, Philadelphia.

Secretary—John M. Swan, Watkins, N. Y.

Treasurer—Charles Lincoln Furbush, Philadelphia.

Assistant Secretary—Edwin C. Shattuck, Philadelphia.

Councillors (to serve two years)—George Dock, New Orleans; Joseph McFarland, Philadelphia; T. B. Futcher, Baltimore.

---

FOR SALE.—The Texas Sanitarium at Llano, Texas. This institution is devoted to the treatment of tuberculosis. It is now well established and enjoys a good patronage. It will pay a good rate of interest on its investment and good salaries to those in charge of its management.

This property offers an exceptional opportunity for two physicians to form a partnership, both receive good salaries and at the same time conduct a joint office in the city of Llano for the practice of medicine and surgery. The price asked for this splendid property will be an inducement to invest, caused by its large shareholders living too far from the institution. For particulars address

DR. M. M. SMITH, Box 207, Dallas, Texas.

---

DR. PETTEY ADMITS PARTNER AND ENLARGES SANITARIUM.—Dr. Geo. E. Pettey, of Memphis, has admitted his former assistant, Dr. W. R. Wallace, as a full partner in his sanitarium and they have built a forty-room addition to their sanitarium.

The new building has been constructed according to plans especially adapted to their work, rooms en suite or single, with private bath, hot and cold water, electric bell system, steam heat with every modern convenience for the employment of hydro- and electrotherapy. Separate buildings for male and female patients.

While their work will be principally confined to the treatment of alcohol and drug addiction they will hereafter also admit patients suffering from mental and nervous diseases. A detached building will be used for the mental cases where special attention will be given to the treatment of acute insanity. Violently insane patients will be admitted for treatment during the acute stage.

THE "PHENOMENAL" COMES BACK AT THE ASSOCIATION.—A telegram from Brenham, Texas, to the *Austin Daily Statesman*, dated May 20 (ult.), says: A suit for $25,000 damages has been filed in the district court of Washington county by Brockmann & Kahn, attorneys for the plaintiff, Dr. J. La Fayette Berry, against the State Medical Association of Texas. The doctor alleges in his petition that he is sixty years of age and has a certificate to practice medicine in Texas and that he has three diplomas from reputable medical colleges in the United States. He further alleges that in its issue of April, 1910, there appeared in the *Texas State Journal of Medicine*, which is managed by the defendants, an article under the heading, "The Passing of the Phenomenal La Fayette," alleged to have been written by James N. Wilkerson, of Fort Worth, general counsel for defendants, and was written by him deliberately and wilfully and for the sole purpose of defaming, ruining and injuring the plaintiff. This suit is the sequel to the prosecution of Dr. Berry at San Antonio, in which his license to practice medicine was revoked, but which case he will appeal to the Court of Appeals.

---

FREE SCHOLARSHIP IN NURSING.—The Philadelphia School for Nurses, 2219 Chestnut Street, Philadelphia, Pa., offers free scholarships in trained nursing to young women in every State in the Union. The scholarships cover the full two years' course, with room, board, uniforms, laundering, etc., included, and railroad fare paid to home town or district upon completion of the course.

A Home Study Course and a Short Resident Course are also provided, which quickly open the door to opportunity and enable progressive students to render a noble service to humanity and at the same time acquire for themselves a substantial income from the best paid occupation now open to women; besides qualifying every student to deal with emergencies in the home that may mean the saving of a loved one's life.

Far-seeing philanthropists are adding to the resources of this school with the view of ultimately extending these benefits to earnest, energetic young women in all country districts and in all the smaller towns and cities.

The institution is approved and endorsed by leading physicians and educators of the entire country. Some of the leading men of this State are its strong supporters and endorsers, as will be seen by the catalogue, which will be sent to anyone who writes to the school for it.

